# Comparison of femoral tunnel length and obliquity of anatomic versus nonanatomic anterior cruciate ligament reconstruction: A meta-analysis

**DOI:** 10.1371/journal.pone.0230497

**Published:** 2020-03-23

**Authors:** Sang-Soo Lee, In-Wook Seo, Min-Soo Cho, Young-Soo Shin

**Affiliations:** 1 Institute for Skeletal Aging & Orthopedic Surgery, Chuncheon Sacred Heart Hospital, Hallym University School of Medicine, Chuncheon, Republic of Korea; 2 Department of Orthopedic Surgery, Veterans Health Service Medical Center, Seoul, Republic of Korea; 3 Department of Orthopedic Surgery, Chuncheon Sacred Heart Hospital, Hallym University School of Medicine, Chuncheon, Republic of Korea; Universitat de Valencia, SPAIN

## Abstract

**Purpose:**

Theoretical considerations suggest that femoral tunnel length might cause graft mismatch, and femoral tunnel obliquity could be related to the longevity of graft in anterior cruciate ligament (ACL) reconstruction. However, controversy still exists regarding these issues in the context of the comparison of anatomic and nonanatomic ACL reconstructions. The purpose of this meta-analysis was to compare the length and obliquity of the femoral tunnel created by drilling through either anatomic or nonanatomic ACL reconstructions.

**Materials and method:**

In this meta-analysis, we reviewed studies that compared femoral tunnel length and femoral tunnel obliquity in the coronal plane with the use of anatomic or nonanatomic ACL reconstruction. The major databases were reviewed for appropriate studies from the earliest available date of indexing through December 31, 2018. No restrictions were placed on the language of publication.

**Results:**

Twenty-seven studies met the criteria for inclusion in this meta-analysis. The femur tunnel length of anatomic ACL reconstruction was significantly shorter compared with that of nonanatomic ACL reconstruction by 8.66 mm (95% CI: 7.10–10.22 mm; P<0.001), while the femur tunnel obliquity in the coronal plane of anatomic ACL reconstruction was significantly more oblique versus that of nonanatomic ACL reconstruction by 15.29° (95% CI: 8.07°–22.52°; P<0.001). Similar results in terms of femoral tunnel length were found for the subgroup with cadaveric (7.15 mm; 95% CI: 2.69–11.61 mm; P = 0.002) and noncadaveric (8.96 mm; 95% CI: 7.24–10.69 mm; P<0.001) studies, whereas different results in terms of femoral tunnel obliquity were noted for the subgroup with cadaveric (10.62°; 95% CI: −6.12° to 27.37°; P = 0.21) and noncadaveric (15.86°; 95% CI: 8.11°–23.60°; P<0.001) studies.

**Conclusion:**

Anatomic ACL reconstruction resulted in the femoral tunnel length and femoral tunnel obliquity in the coronal plane being shorter and more oblique, respectively, as compared with nonanatomic ACL reconstruction.

**Level of evidence:**

Therapeutic study, Level III.

## Introduction

The goal of anterior cruciate ligament (ACL) reconstruction is to provide the patient with a graft that replicates the normal kinematics of the knee.[1,2] As a result of the desire to reproduce normal kinematics of the knee during the creation of the ACL femoral tunnel, arthroscopic ACL reconstruction has evolved from a transtibial (TT) technique to anteromedial (AM) portal or outside-in (OI) technique despite the fact that OI technique mostly predated TT technique; TT technique evolved from OI technique as a way to perform anthroscopic ACL reconstructions more easily. OI technique came back into favor and AM portal technique became more popular with an attempt to place the femoral tunnel within the footprint.[3–5] Thus, most studies have focused predominantly on illustrating the femoral tunnel location in order to show the superiority of reproducing ACL footprints in anatomical reconstructions in comparison with those in TT techniques. However, femoral tunnel length and femoral tunnel obliquity have clinically important relevance because the former might also cause the problem of graft mismatch and the latter could be related to graft longevity.[6–8] It has been well-established through previous studies that the AM portal or OI technique, one of the anatomic ACL reconstruction options, results in a shorter femoral tunnel than does the TT technique.[9,10] These findings are of clinical relevance in that such can lead to the problem of graft incorporation and stability due to a reduced length of graft within the femoral tunnel at the time of reconstruction.[11,12] In addition, biomechanical studies have supported a more oblique femoral tunnel position in the coronal plane because of a decrease in tension across the graft, increased range of motion, and reduced posterior cruciate ligament (PCL) impingement.[13,14] Also, one previous study on the association between graft-bending angle and computed tomography (CT) plane found that OI technique had a significantly larger coronal bending angle than the AM portal technique, but not in the axial and sagittal planes.[10] Importantly, controversy still exists regarding femur tunnel obliquity with respect to the results of measurement values between anatomic and nonanatomic ACL reconstructions. Although many studies have been published to date focusing on single-bundle and double-bundle ACL reconstruction, few comparative studies assessing femoral tunnel length and femoral tunnel obliquity between anatomic and nonanatomic ACL reconstructions have been completed at this time.

It is still controversial which of these methods is appropriate to achieve proper femoral tunnel length and obliquity. In addition, investigation of these parameters was deemed to be important because they could determine the longevity of graft in ACL reconstruction. The purpose of this meta-analysis was to determine the length of the femoral tunnel created by drilling through either anatomic or nonanatomic ACL reconstructions. Additionally, we sought to determine the obliquity of a femoral tunnel when placed through anatomic or nonanatomic ACL reconstructions.

## Materials and methods

This meta-analysis was conducted according to the guidelines of the preferred reporting items for systematic reviews and meta-analysis (PRISMA) statement (S1 PRISMA Checklist).

### Data and literature sources

We performed an electronic records search in the MEDLINE (Inception to December 2018), EMBASE (Inception to December 2018), Cochrane Library (Inception to December 2018), and KoreaMed (Inception to December 2018) databases.

### Study selection

Based on the title and abstract, two reviewers independently selected relevant studies for further review. Each reviewer reviewed one database, which in turn was validated twice by the other reviewer. The full text of selected studies was analyzed if the abstract did not provide enough data to make a decision. Only studies comparing anatomic single-or double-bundle ACL reconstruction versus nonanatomic single-or double-bundle ACL reconstruction were included in this meta-analysis, regardless of graft type or fixation method. Anatomic ACL reconstruction was defined as a technique having the intra-articular opening of the femoral tunnel created by independent drilling such as the AM portal and OI techniques which may lie inside the true femoral footprint of the ACL. Non-anatomic ACL reconstruction was defined as a technique having the intra-articular opening of the femoral tunnel created by the TT technique which may lie outside the true femoral footprint of the ACL because of constraints in the direction of the tibial tunnel. Primary outcomes that were recorded included femoral tunnel length. Secondary outcomes included femoral tunnel obliquity in the coronal plane.

After eliminating duplicate results, studies were included in the meta-analysis if they (1) evaluated knees previously undergone anatomic ACL reconstruction or nonanatomic ACL reconstruction; (2) had an evidence level of 1 (high quality randomized trial or prospective study) or 2 (lesser quality randomized controlled trial or prospective comparative study) or 3 (case control study or retrospective comparative study); (3) reported a retrospective or prospective comparison of anatomic ACL reconstruction and nonanatomic ACL reconstruction cohorts; (4) included data of at least one of the following two knee joint parameters: femoral tunnel length and femoral tunnel obliquity. Femoral tunnel obliquity was calculated only in the coronal plane because insufficient detail in reporting prevented valid calculation of the effective size. Additionally, studies were included if they (5) fully reported the number of subjects in each group and the means and standard deviations for the two parameters; and (6) used adequate statistical methods to compare these parameters between groups. conversely, studies were excluded if they (1) did not meet the inclusion criteria; if they (2) had missing or inadequate outcomes data, such as standard deviations or ranges of values; or (3) were case series, expert opinions, reviews, commentaries, or editorials.

### Data extraction

Two reviewers recorded data from each study using a predefined data extraction form and resolved any differences by discussion. The data were extracted for each study: (1) author identification; (2) year of publication; (3) study design and methodological quality information needed to complete the Cochrane Collaboration's tool for assessing risk of bias; (4) sample size; (5) inclusion/exclusion criteria; (6) baseline characteristics; (7) surgical outcomes used such as femoral tunnel length and femoral tunnel obliquity in the coronal plane for patients with either anatomic ACL reconstruction or nonanatomic ACL reconstruction and (8) duration of follow-up.

### Methodological quality assessment

Two reviewers independently assessed the methodological quality of the studies. For the Newcastle–Ottawa Scale, as recommended by the Cochrane Nonrandomized Studies Methods Working Group, we assessed studies based on the following three criteria: selection of the study groups, comparability of the groups, and ascertainment of either the exposure or the outcome of interest for case-control and cohort studies. Studies of high quality were defined as those with scores higher than six points. Two reviewers resolved all differences by discussion, and their decisions were subsequently reviewed by a third investigator.

### Data synthesis and analysis

If a study presented a different surgical technique for the anatomic ACL reconstruction, data from the different surgical technique were analyzed as separate studies. If these variables were not included in the articles, the weighted mean difference was calculated from the p-value and sample size. Meta-analysis was performed using the Revman 5.3 software with a random-effects model, which was used to account for heterogeneity, and the Stata version 14.2 static software. The main outcomes of the meta-analysis were the weighted mean difference (WMD) in femoral tunnel length and femoral tunnel obliquity in the coronal plane. For all comparisons, WMD values and 95% CIs were calculated for continuous outcomes. Heterogeneity was determined by estimating the proportion of between-study inconsistencies due to actual differences between studies, rather than differences due to random error or chance. I^2^ statistics with a value of less than 40% represents low heterogeneity and a value of 75% or more indicates high heterogeneity. When statistical heterogeneity was substantial, we conducted a meta-regression to identify potential sources of bias such as time from surgery to image and measurement tools. The risk of bias (e.g., low, high, or unclear) was independently assessed by two investigators. Publication bias was also assessed using funnel plots and Egger’s test. Subgroup analyses based on the presence or absence of human knees were performed for two endpoints to explore a potential source of heterogeneity. As a result, each group was divided into two subgroups: cadaver and noncadaver. Additionally, sensitivity analysis was performed by excluding eligible studies one at a time. Studies with data from the outside-in technique, double bundle technique, or using a flexible reamer were included, while other studies with a different study type were included. Pooling of data was feasible for the following two outcomes of interest: femoral tunnel length and femoral tunnel obliquity.

## Results

### Study identification, study characteristics, patient population, quality assessment, and publication bias of the included studies

**Fig 1:**
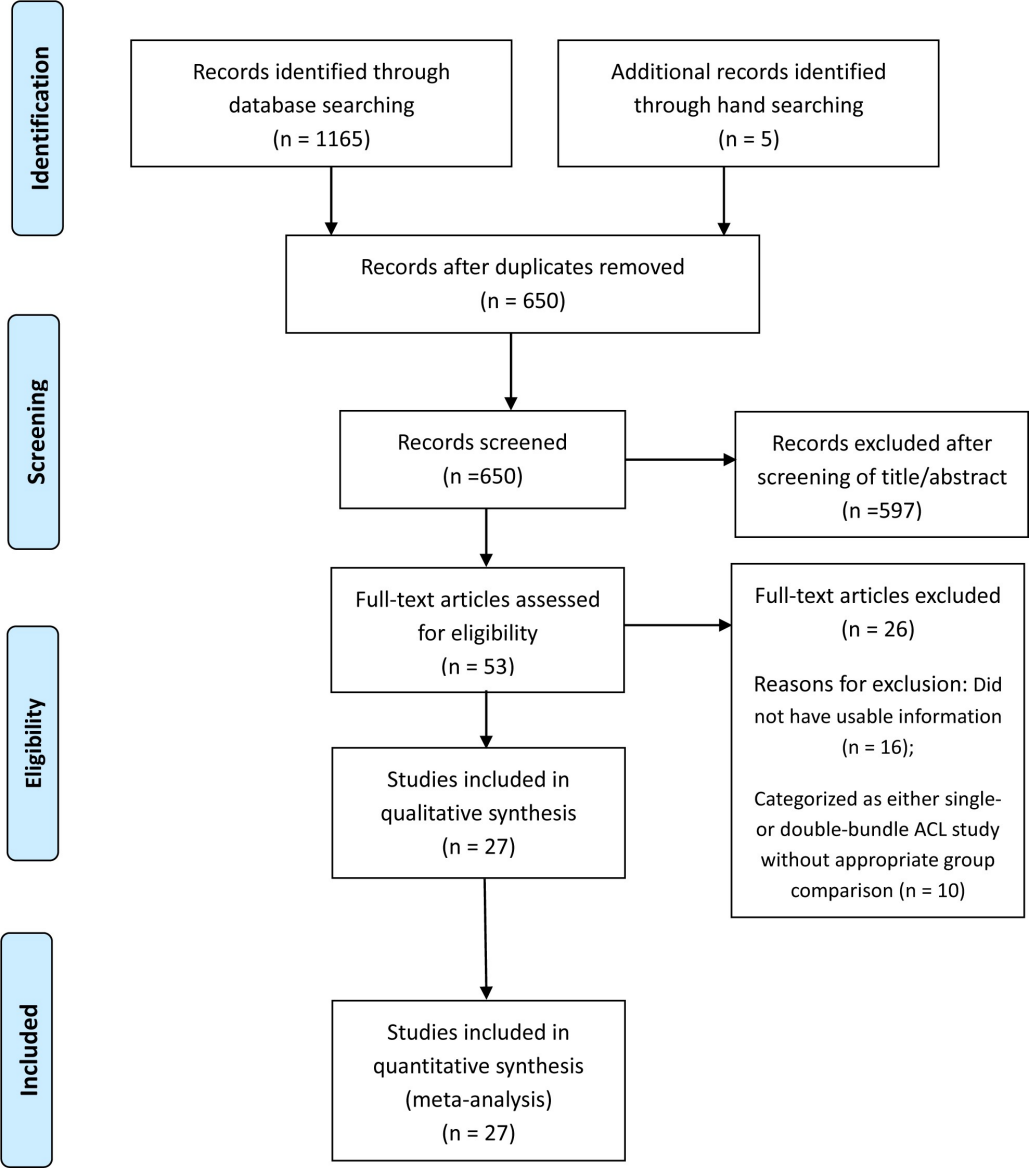
A flow diagram of Preferred Reporting Items for Systemic Reviews and Meta-Analyses (PRISMA).

**Fig 2:**
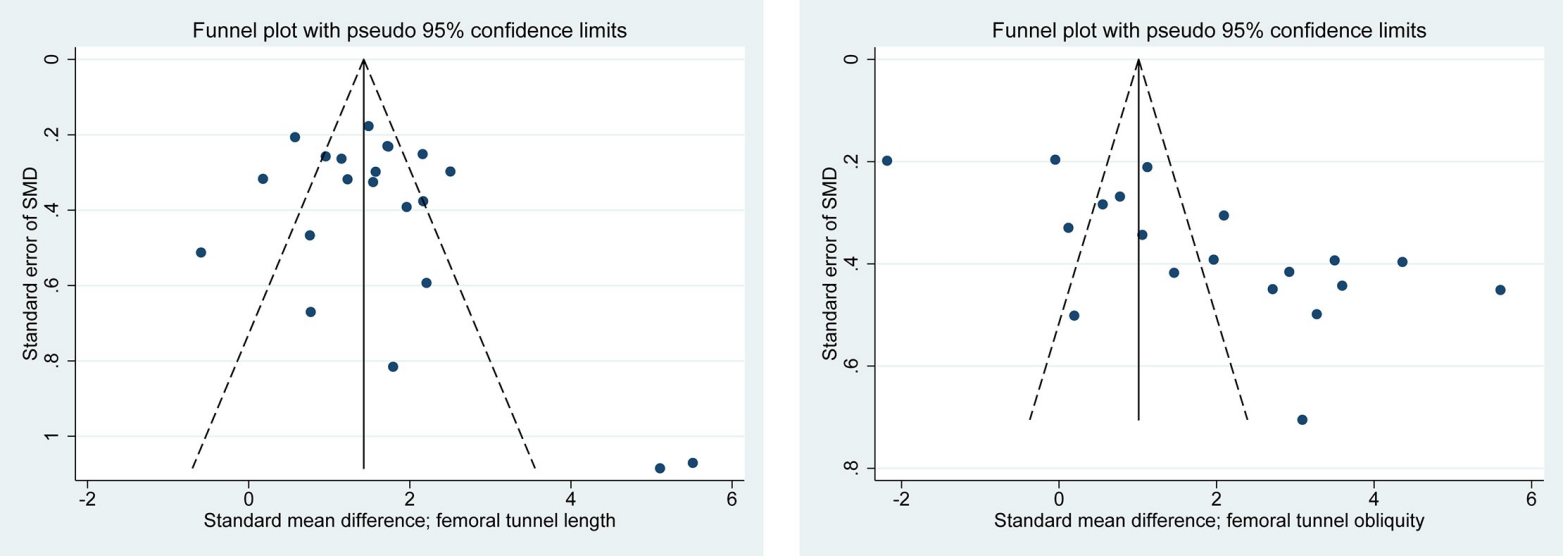
Funnel plot showing relatively symmetric data on femoral tunnel length **(A)** between patients with anatomic and non-anatomic ACL reconstruction, suggesting lack of publication biases. However, funnel plot showing asymmetric data on femoral tunnel obliquity **(B)** between patients with anatomic and non-anatomic ACL reconstruction, suggesting some publication bias among included studies.

Fig 1 shows details of study identification, inclusion, and exclusion. Ultimately, 27studies were included in this meta-analysis. The 27 studies [15–38] included a total of 1,693 subjects (anatomic ACL reconstruction: 971 subjects; nonanatomic ACL reconstruction: 722 subjects). Characteristics of the included studies are described in Table 1. Quality findings of the 27 studies included in the meta-analysis are summarized in Table 1. The non–randomized controlled trials (15 PCSs and 12 RCSs) were of high quality (Newcastle–Ottawa Scale > 6). Inter-rater reliabilities (k values) for all items of the Newcastle–Ottawa Scale ranged from 0.78 to 0.89, suggesting at least more than substantial agreement between the two investigators. We evaluated the publication bias of femoral tunnel length and femoral tunnel obliquity. Funnel plots showed that the mean differences in femoral tunnel length were relatively symmetric (Fig 2A), indicating a lack of publication bias among the included studies. However, mean differences in femoral tunnel obliquity were skewed right asymmetrically, indicating some publication bias among the included studies (Fig 2B). Egger’s test confirmed these trends of publication bias in femoral tunnel length (P = 0.297) and femoral tunnel obliquity (P = 0.001), respectively.

**Table 1:** Summary of patient characteristics of the included studies.

Study	Year	Study type	Sample size (M/F)		Age		Evaluation method	Time from surgery to image	Femoral drilling technique/Graft type (SB/ DB(AM))	Cadaver or Noncadaver	Quality score	Measured parameters
			Non-anatomical	Anatomical AM OI	Non-anatomical	Anatomical AM OI						
Hanteset al.[19]	2009	RCS	30 (NA)	26 (NA)	25.6	27.2	MRI	1Yr	TT(n = 30), AMP(n = 26)/(SB)	Noncadaver	NOS 7	Tunnel obliquity
Bediet al.[16]	2010	PCS	6 (NA)	12 (NA)	NA	NA	CT	Immediate postoperatively	TT(n = 6), AMP(n = 12)/(SB)	Cadaver	NOS 7	Tunnel length, Tunnel obliquity
Bower et al.[17]	2010	PCS	15 (NA)	15 (NA)	24(16–28)	24(16–28)	MRI	12weeks	TT(n = 15), AMP(n = 15)/(SB)	Noncadaver	NOS 7	Tunnel obliquity
Miller et al.[26]	2011	PCS	10 (NA)	10 (NA)	73–89		CT	Immediate postoperatively	TT(n = 10), AMP(n = 10)/(SB)	Cadaver	NOS 8	Tunnel length
Chang et al.[9]	2011	RCS	55 (39/16)	50 (39/11)	31.8±11.7	31.9±11.7	Intraoperative depth gauge, Plain radiograph	Immediate postoperatively	TT(n = 55), AMP(n = 50)/(SB)	Noncadaver	NOS 7	Tunnel length, Tunnel obliquity
Wang et al.[38]	2012	PCS	20 (NA)	29 (NA0)	32.7±9	32±10.8	CT	NA	TT(n = 20)/(SB), AMP(n = 29)/(DB)	Noncadaver	NOS 7	Tunnel length
Ilahiet al.[21]	2012	RCS	35 (NA)	80 (NA)	24.4 (14–45)	23.2 (14–46)	Intraoperative depth gauge	Intraoperatively	TT(n = 35), AMP(n = 80) /(SB)	Noncadaver	NOS 8	Tunnel length
Larson et al.[23]	2012	PCS	5 (NA)	10 (NA)	71(49–91)		CT	Immediate postoperatively	TT(n = 5), AMP(n = 5), OI(n = 5) /(SB)	Cadaver	NOS 7	Tunnel length
Hensleret al.[20]	2013	PCS	27 (NA)	20 (NA)	NA	NA	CT	Immediate postoperatively	TT(n = 27), AMP(n = 20) /(SB)	Noncadaver	NOS 7	Tunnel length
Takeda et al.[34]	2013	PCS	25 (19/6)	25 (19/6)	27.8 (15–48)	27.7 (15–47)	CT	1 week	TT(n = 25), AMP(n = 25) /(DB)	Noncadaver	NOS 7	Tunnel length, Tunnel obliquity
Tompkinset al.[37]	2013	PCS	10 (NA)	10 (NA)	74.9±11.3		CT	Immediate postoperatively	TT(n = 10), AMP(n = 10) /(SB)	Cadaver	NOS 7	Tunnel length, Tunnel obliquity
Pascual-Garrido et al.[1]	2013	RCS	17 (NA)	23 (NA)	NA	NA	Plain radiograph	1 week	TT(n = 17), AMP(n = 23) /(SB)	Noncadaver	NOS 7	Tunnel obliquity
Shin et al.[10]	2014	RCS	37 (32/5)	82 (49/23) 46 (37/9)	35 (19–60)	30 (15–53) 32 (16–60)	CT	Immediate postoperatively	TT(n = 37), AMP(n = 82), OI(n = 46) /(SB)	Noncadaver	NOS 7	Tunnel length
Lee et al.[25]	2014	RCS	52 (NA)	52 (NA)	NA	NA	CT	Immediate postoperatively	TT(n = 52), AMP(n = 52) /(SB)	Noncadaver	NOS 7	Tunnel length, Tunnel obliquity
Song et al.[33]	2014	PCS	30 (NA)	30 (NA)	NA	NA	CT	2–6 weeks	TT(n = 30), AMP(n = 30) /(SB)	Noncadaver	NOS 7	Tunnel length
Seoet al.[29]	2014	RCS	41 (32/9)	48 (40/8)	30.6±11.14	32.4±13.3	Plain radiograph	Immediate postoperatively	TT(n = 41), OI(n = 48) /(SB)	Noncadaver	NOS 7	Tunnel obliquity
Sohn et al.[32]	2014	RCS	20 (19/1)	20 (19/1) 20 (17/3)	29.5 (16–46)	26.9 (17–49) 31.4 (15–51)	Plain radiograph	Immediate postoperatively	TT(n = 20), AMP(n = 20), OI(n = 20) /(SB)	Noncadaver	NOS 7	Tunnel obliquity
Arcuri et al.[15]	2014	PCS	19 (NA)	40 (NA)	NA	NA	Plain radiograph	NA	TT(n = 19), AMP(n = 40) /(SB)	Noncadaver	NOS 7	Tunnel obliquity
Sirleoet al.[31]	2014	PCS	20 (NA)	20 (NA)	NA	NA	CT	NA	TT(n = 20), OI(n = 20) /(SB)	Noncadaver	NOS 7	Tunnel obliquity
Celiktaset al.[18]	2015	PCS	81 (NA)	83 (NA)	29.6±8.4		Plain radiograph	NA	TT(n = 81), AMP(n = 83) /(SB)	Noncadaver	NOS 7	Tunnel length, Tunnel obliquity
Ostiet al.[28]	2015	PCS	36 (20/16)	32 (27/5) 32(19/13)	33.59±12.17	31.2±10.64 33.74±7.52	CT	Immediate postoperatively	TT(n = 36), AMP(n = 32), OI(n = 32) /(SB)	Noncadaver	NOS 7	Tunnel length, Tunnel obliquity
Tasdemiret al.[36]	2015	RCS	15 (13/2)	24 (20/4)	29.73±6.33	29.04±7.53	MRI	2Yrs	TT(n = 15), AMP(n = 24) /(SB)	Noncadaver	NOS 6	Tunnel obliquity
Shetty et al.[30]	2016	RCS	30 (NA)	30 (NA)	NA	NA	Plain radiograph	Immediate postoperatively	TT(n = 30), AMP(n = 30) /(SB)	Noncadaver	NOS 7	Tunnel obliquity
Jennings et al.[22]	2017	PCS	6 (NA)	12 (NA)	NA	NA	Intraoperative depth gauge	Intraoperatively	TT(n = 6), AMP(n = 12) /(SB)	Cadaver	NOS 7	Tunnel length
Nakamura et al.[27]	2018	RCS	20 (12/8)	20 (12/8) 20 (15/5)	26.2 ± 6.2	26.4 ± 5.2 25.3 ± 3.1	Intraoperative depth gauge	Intraoperatively	TT(n = 20), AMP(n = 20), OI(n = 20) /(DB)	Noncadaver	NOS 8	Tunnel length
Tampere et al.[35]	2018	PCS	10 (5/5)	10 (7/3)	27(21–37)	30.5(20–40)	CT	NA	TT(n = 10), AMP(n = 10) /(SB)	Noncadaver	NOS 7	Tunnel length
Lee et al.[24]	2018	RCS	50 (34/16)	50 (32/18)	28.0±5.6	27.2±7.3	CT	Immediate postoperatively	TT(n = 50), OI(n = 50) /(SB)	Noncadaver	NOS 7	Tunnel obliquity

Abbreviations: RCS, retrospective comparative study; PCS, prospective comparative study; M, male; F, female; NA, not available; NOS, Newcastle-Ottawa Scale; CT, computed tomography; MRI, magnetic resonance imaging; TT, transtibial; AMP, anteromedial portal; OI, outside-in; SB, Single bundle; DB, Double bundle; AM, Anteromedial

### Femoral tunnel length

**Fig 3:**
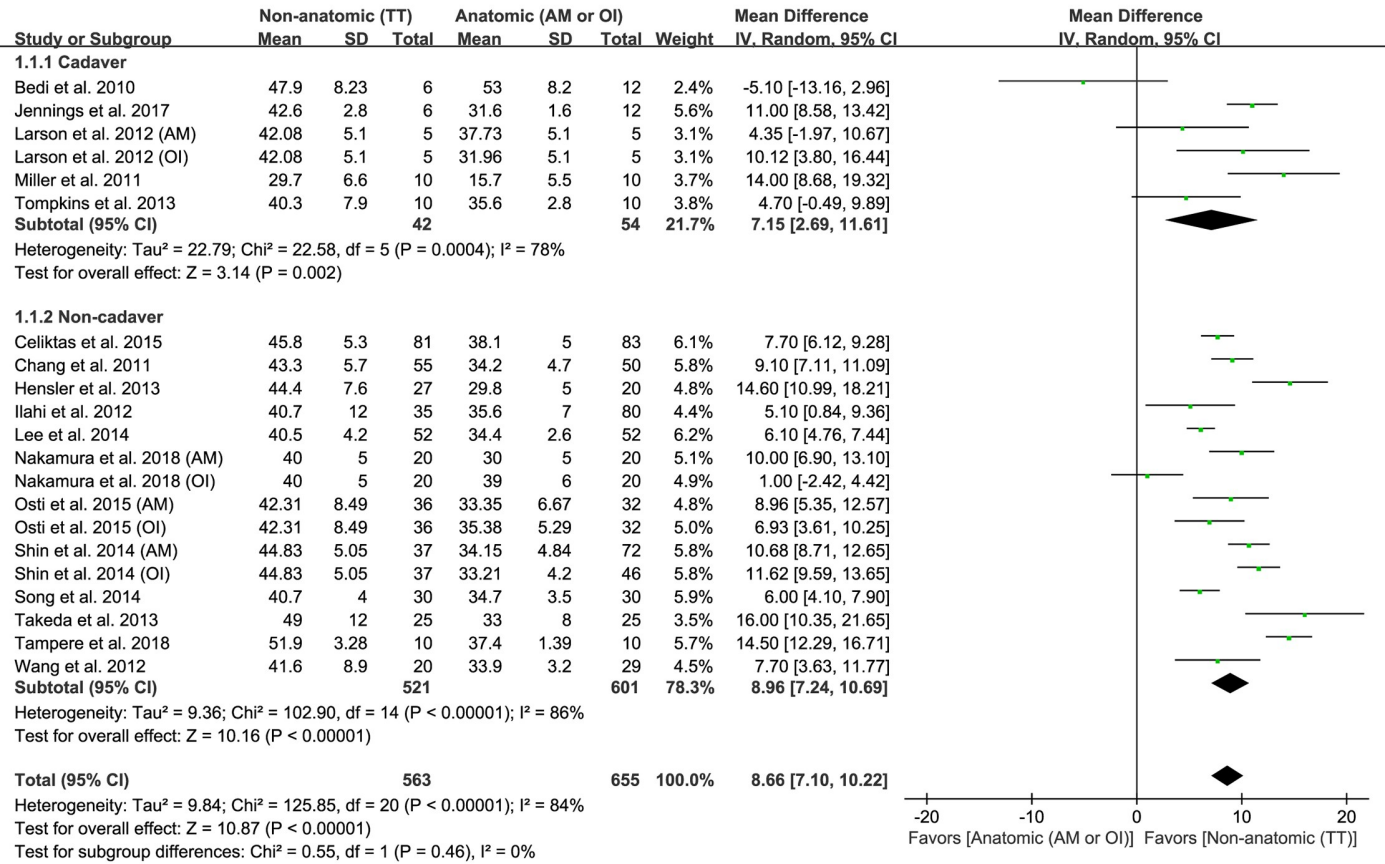
Results of aggregate analysis for comparison of femoral tunnel length between patients with anatomic and non-anatomic ACL reconstruction, including subgroup analysis by cadaveric and non-cadaveric studies.

Of the 27 studies, 21 reporting on femoral tunnel length were included. Six hundred fifty-five subjects were operated on using anatomic ACL reconstruction and 563 subjects underwent nonanatomic ACL reconstruction. The pooled data revealed that the mean difference in femoral tunnel length was 8.66 mm (95% CI: 7.10–10.22 mm; P<0.001; I^2^ = 84%; Fig 3), indicating that femoral tunnel length was significantly greater in nonanatomic ACL reconstruction than in anatomic ACL reconstruction. Six studies were assigned to the cadaver subgroup and 15 studies were assigned to the noncadaver subgroup. The cadaver subgroup demonstrated a significantly greater femoral tunnel length by 7.15 mm (95% CI: 2.69–11.61mm; P = 0.002; I^2^ = 78%; Fig 3) in nonanatomic ACL reconstruction than in anatomic ACL reconstruction. Similarly, the noncadaver subgroup showed a significantly greater femoral tunnel length by 8.66 mm (95% CI: 7.24–10.69 mm; P<0.001; I^2^ = 86%; Fig 3) in nonanatomic ACL reconstruction than in anatomic ACL reconstruction. Based on the results of sensitivity analysis, a statistical difference could not be shown as compared with the results of the original analysis, suggesting that the findings are robust in the context of decisions made in their collection process (Table 2).

**Table 2:** Sensitivity analysis.

Study	Parameter	Before exclusion	After exclusion	Statistical significance
TT vs OI	femoral tunnel length	MD = 8.66, 95% CI = 7.10,10.22, Z = 10.87, P< 0.001	MD = 8.98, 95% CI = 7.30, 10.66, Z = 10.49, P< 0.001	No difference
	femoral tunnel obliquity	MD = 15.29, 95% CI = 8.07, 22.52, Z = 4.15, P< 0.001	MD = 13.19, 95% CI = 5.71, 20.67, Z = 3.46, P< 0.001	No difference
DB	femoral tunnel length	MD = 8.66, 95% CI = 7.10,10.22, Z = 10.87, P< 0.001	MD = 8.81, 95% CI = 7.38, 11.05, Z = 10.52, P< 0.001	No difference
	femoral tunnel obliquity	MD = 15.29, 95% CI = 8.07, 22.52, Z = 4.15, P< 0.001	MD = 14.97, 95% CI = 7.42, 22.52, Z = 3.89, P< 0.001	No difference
Flexible reamer	femoral tunnel length	MD = 8.66, 95% CI = 7.10,10.22, Z = 10.87, P< 0.001	MD = 8.52, 95% CI = 6.89, 10.15, Z = 10.24, P< 0.001	No difference
RCS	femoral tunnel length	MD = 8.66, 95% CI = 7.10,10.22, Z = 10.87, P< 0.001	MD = 9.11, 95% CI = 6.95, 11.28, Z = 8.24, P< 0.001	No difference
	femoral tunnel obliquity	MD = 15.29, 95% CI = 8.07, 22.52, Z = 4.15, P< 0.001	MD = 15.21, 95% CI = 2.33, 28.09, Z = 2.31, P = 0.02	No difference

TT, transtibial; OI, outside-in; DB, double bundle; RCS, Retrospective comparative study; CI, confidence interval; MD, mean difference

### Femoral tunnel obliquity

**Fig 4:**
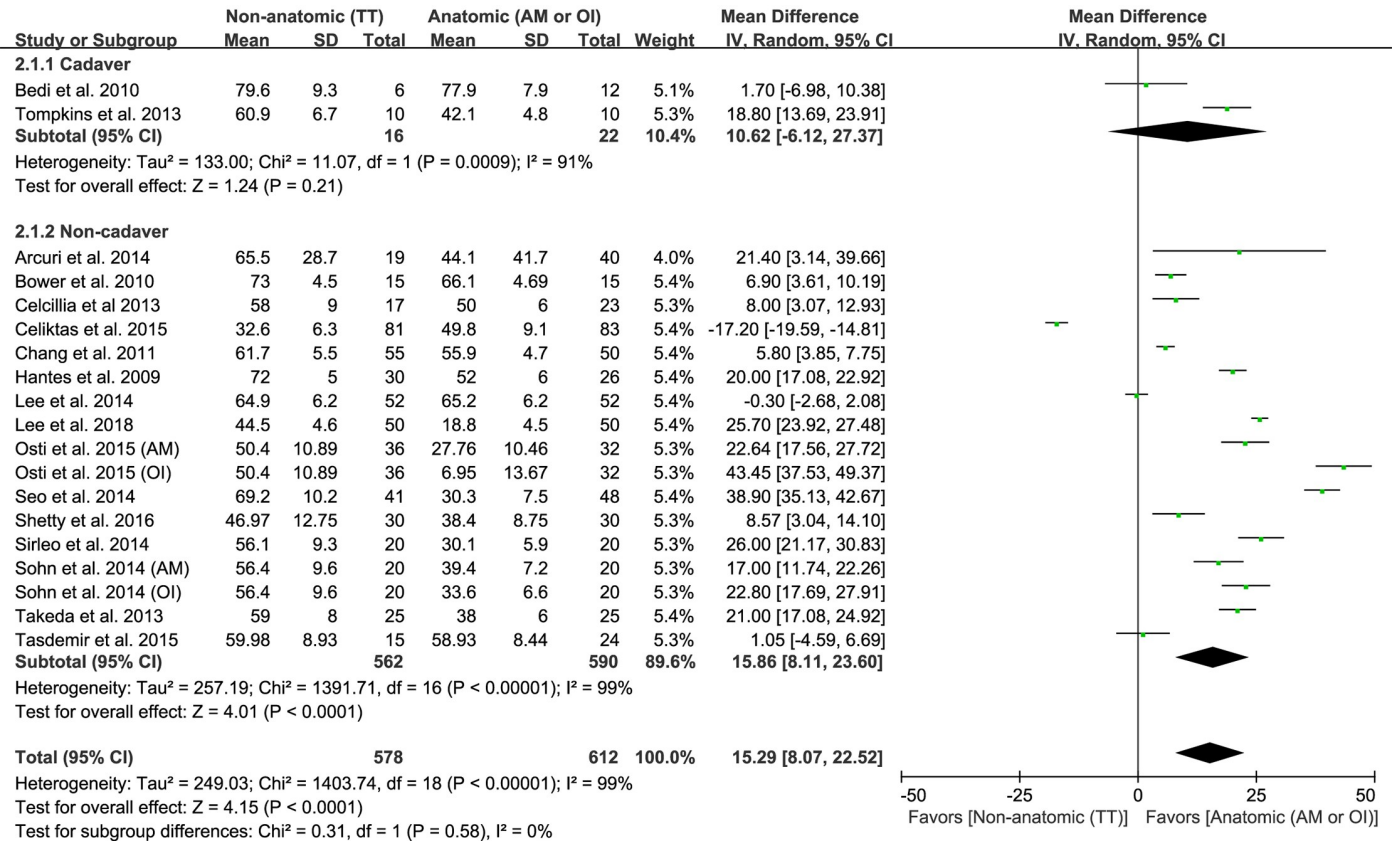
Results of aggregate analysis for comparison of femoral tunnel obliquity between patients with anatomic and non-anatomic ACL reconstruction, including subgroup analysis by cadaveric and non-cadaveric studies.

Of the 27 studies, 19 reporting on femoral tunnel obliquity in the coronal plane were included. Six hundred twelve subjects were operated on using anatomic ACL reconstruction and 578 subjects were operated on using nonanatomic ACL reconstruction. The pooled data showed that the mean femoral tunnel obliquity difference was 15.29° (95% CI: 8.07°–22.52°; P<0.001; I^2^ = 99%; Fig 4), indicating that the femur tunnel obliquity of anatomic ACL reconstruction was significantly more oblique as compared with that of nonanatomic ACL reconstruction. For subgroup analysis, two studies were assigned to the cadaver subgroup, while 17 studies were assigned to the noncadaver subgroup. In the cadaver subgroup, the anatomic ACL reconstruction led to 10.62° more obliquity than did nonanatomic ACL reconstruction, but this difference was not significant (95% CI: −6.12° to 27.37°; P = 0.21; I^2^ = 91%; Fig 4). In contrast, the pooled mean difference in the noncadaver subgroup was 15.86° (95% CI: 8.11°–23.60°; P<0.001; I^2^ = 99%; Fig 4). The results of sensitivity analysis were not significantly different from those of the original analysis, including that the findings are robust in terms of the decisions made in the process of obtaining them (Table 2).

### Meta-regression analysis

The results of the meta-regression analysis are summarized in Table 3. For femoral tunnel length, between the two groups, we did not identify the time from surgery to image (P = 0.153) and measurement (P = 0.886) tool application as a source of heterogeneity. Similarly, we did not identify the time from surgery to image (P = 0.632) and measurement (P = 0.445) tool application as sources of heterogeneity for femoral tunnel obliquity between the two groups.

**Table 3:** Meta-regression analyses of potential sources and difference in femoral tunnel length or femoral tunnel obliquity for anatomic and non-anatomic ACL reconstruction.

Variable	Coefficient	Standard error	P value	95% confidence interval
Femoral tunnel length				
Time from surgery to image, weeks (≤12 or ≥12)	0.998	0.666	0.153	-0.413 to 2.410
Measurement tools (CT and MRI or Others)	-0.082	0.565	0.886	-1.264 to 1.100
Femoral tunnel obliquity				
Time from surgery to image, weeks (≤12 or ≥12)	-0.526	1.074	0.632	-2.829 to 1.776
Measurement tools (CT and MRI or Others)	-0.675	0.862	0.445	-2.494 to 3.459

CT, computed tomography; MRI, magnetic resonance imaging

## Discussion

The principal findings from this meta-analysis were that anatomic ACL reconstruction resulted in the femoral tunnel length and femoral tunnel obliquity being shorter and more oblique, respectively, than nonanatomic ACL reconstruction as hypothesized.

The AM portal and OI technique is able to shorten the available femoral tunnel length because of a shorter distance between the starting point and the lateral femoral cortex.[9,10,39,40] This shortened femoral tunnel may be associated with a lower pull-out strength and decreased graft healing as the graft has less grip on the short tunnel even though it has been investigated in other studies that 15–20 mm is plenty of pull-out strength.[41] For example, one study compared the length of the femoral tunnel created by either a TT or AM portal technique using 10 matched-pair fresh-frozen cadaveric knees. That study determined that shorter femoral tunnel lengths were observed when drilling through an AM portal versus using the TT technique.[26] These results confirm those of an earlier study, in which the length of femoral tunnel after anatomic ACL reconstruction techniques, including both the AM and OI techniques, was shorter than that after using the TT technique.[10] In contrast, another investigation showed quite shorter tunnels than in the studies quoted previously with the TT technique, which can cause higher heterogeneous results, leading to an inconclusive meta-analysis.[25] This indicates that the starting position of the tibial tunnel has an impact on the femoral tunnel length, demonstrating that using a more medially located tibial tunnel between the midpoint and the posteromedial tibia allows for the creation of a shorter femoral tunnel although this may allow for a more anatomic femoral tunnel placement of the graft. However, our subgroup analysis findings that evaluated mean femoral tunnel length suggested that the femoral tunnel length was significantly greater in nonanatomic ACL reconstruction versus anatomic ACL reconstruction, regardless of the presence or absence of human knees. This discrepancy may be attributable to the fact that more oblique femoral tunnels in the coronal plane created via a far medially locatedtibial tunnel were less pronounced than expected. In addition, research with the knee flexed to 120° found quite shorter tunnels than in the studies quoted previously that used the AM portal technique. These findings differ from previous reports that knee hyperflexion is required when reaming the femoral tunnel through the AM portal to avoid short tunnels.[16] This indicates that different knee flexion angles in the AM portal technique might also be potential reasons for varying results. However, this also may be different if a flexible reamer was used which can allow for a greater femoral tunnel length.[22,23] Our findings from sensitivity analysis evaluating the use of flexible reamer for femoral tunnel length showed that the mean difference of femoral tunnel lengths are 8.66 mm (before exclusion) and 8.52 mm (after exclusion), respectively, but these differences were not significant. Furthermore, it is possible that the measured flexion angle was effectively less than 110° due to being covered by a tourniquet. Together, these facts clinically suggest that the accurate starting position of the tibial tunnel with the TT technique and knee hyperflexion of far greater than 120° with the AM portal technique may have advantages during ACL reconstruction to prevent shorter femoral tunnels.

Many studies have investigated femoral tunnel obliquity in patients who underwent conventional TT and anatomic ACL reconstruction. In biomechanical investigations, an oblique femoral tunnel position in the coronal plane improves rotatory stability as compared with a more vertical location.[16,42] Similarly, the current meta-analysis found that using anatomic ACL reconstruction yields a significantly more oblique femoral tunnel in comparison with the nonanatomic ACL reconstruction. However, one study acknowledged that it is possible for the surgeon to place the femoral tunnel at 60° in the coronal plane during nonanatomic ACL reconstruction if the surgeon maneuvers the angle of the tibial tunnel.[25] To obtain these better results, the entry point of the tibial tunnel must be placed more medially between the midpoint and the posteromedial point and start close to the joint line. Of 19 studies with nonanatomic ACL reconstruction, three [16,17,19] revealed that the angle of the femoral tunnel in the coronal plane was greater than 70°. These different results may be caused by a less-standard starting point of the tibial tunnel, which remained compromised with nonanatomic ACL reconstruction. In contrast, of the 19 studies with anatomic ACL reconstruction, only two [24,28] demonstrated that the angle of the femoral tunnel in the coronal plane was less than 30°, thus showing a much more oblique tunnel than in the studies quoted previously that used anatomic ACL reconstruction. Therefore, a considerably oblique femoral tunnel angle, which may result in more repetitive bending stress on the graft at the femoral tunnel opening, should be avoided because of increased abrasive force at the contact area on the sharp edge of the bone tunnel opening.[7,38]

This study had several limitations. First is that we excluded all studies concerning ACL reconstruction with clinical outcomes and only compared certain examples of anatomical ACL reconstruction such as the AM portal and OI techniques due to the nonavailability of data for assessing femoral tunnel length and femoral tunnel obliquity of anatomic or nonanatomic ACL reconstruction. However, these studies, which tended to be in larger populations, are also of importance. Therefore, this may distort the outcomes if they were put together. The second for improvement involves pooling very heterogeneous data (single and double bundle ACL reconstructions, different imaging modalities used to determine the outcomes, AM and OI techniques mixed together in the anatomic group, cadaveric versus non-cadaveric studies, and levels I, II, and III studies), which are reflected by I^2^ values of the various analyses, although we used a random effects model, subgroup analyses, sensitivity analysis, and meta-regression analysis to incorporate heterogeneous outcomes. However, we still chose these outcomes. Therefore, this should be contemplated when one is interpreting our findings. An additional weakness is that we included one study [22] using a novel flexible reamer that is a significant source of heterogeneity requiring future expansion even though it could not affect the femoral tunnel length in our study.

## Conclusions

Anatomic ACL reconstruction resulted in the femoral tunnel length and femoral tunnel obliquity in the coronal plane being shorter and more oblique, respectively, as compared with nonanatomic ACL reconstruction.

## Supporting information

S1 Checklist PRISMA checklist. (DOC)

S1 Appendix (DOCX)
