# Supplementary material for: Comparison of femoral tunnel length and obliquity of anatomic versus nonanatomic anterior cruciate ligament reconstruction: A meta-analysis
Source: PLoS One. 2020 Mar 23;15(3):e0230497. doi: 10.1371/journal.pone.0230497 (PMC7089554; doi:10.1371/journal.pone.0230497)
Supplement: S1 Appendix — (DOCX) [file pone.0230497.s002.docx]

The following search terms were looked for in the title, abstract, and keyword fields: (((((("tunnel placement"[tiab] OR "femoral tunnel"[tiab] OR "tunnel position"[tiab])) OR ("tunnel length"[tiab] OR "tunnel obliquity"[tiab]))) AND (anteromedial[tiab] OR outside-in[tiab] OR transtibial[tiab] OR transportal[tiab]))) AND (((((("anterior cruciate ligament/anatomy and histology"[Mesh] OR "anterior cruciate ligament/surgery"[Mesh]))) OR (("femur/anatomy and histology"[Mesh] OR "femur/surgery"[Mesh])))) OR (("anterior cruciate ligament reconstruction"[Mesh]) OR ("anterior cruciate ligament reconstruction"[tiab] OR "ACL reconstruction"[tiab]))). There were no restrictions on language. We also performed a hand search for articles potentially missed by the electronic database search.
